# Prosthetic valve obstruction: the ‘frozen leaflet sign’ on routine chest radiography

**DOI:** 10.1093/ehjcr/ytag126

**Published:** 2026-02-23

**Authors:** Jiao Bai, Xiaojun Xie

**Affiliations:** Department of Radiology, The Affiliated Hospital of Southwest Medical University, 8 Kangcheng Road, Jiangyang District, Luzhou 646000, China; Department of Cardiovascular Surgery, The Affiliated Hospital of Southwest Medical University, 8 Kangcheng Road, Jiangyang District, Luzhou 646000, China; Key Laboratory of Medical Electrophysiology, Ministry of Education & Medical Electrophysiological Key Laboratory of Sichuan Province, Luzhou 646000, China

**Keywords:** Prosthetic valve obstruction, Valve replacement, Chest radiography

## Summary

This case report describes a 52-year-old female with a history of mechanical mitral valve replacement who presented with acute decompensated heart failure. A portable chest X-ray revealed a linear radiopaque density aligned with the prosthetic annulus, which we term the ‘frozen leaflet sign’, suggesting possible leaflet immobilization. Transthoracic echocardiography confirmed elevated transprosthetic gradients and severe pulmonary hypertension. Despite a therapeutic INR on admission, acute prosthetic valve thrombosis was suspected and later confirmed during urgent surgery, which revealed thrombotic occlusion of the lateral leaflet. Successful redo valve replacement led to the resolution of radiographic abnormalities. This case illustrates that the ‘frozen leaflet sign’ on routine chest radiography can be a suggestive clue to prosthetic valve obstruction, potentially aiding in early suspicion, particularly in settings where advanced imaging may not be immediately accessible.

## Case description

**Figure 1.**
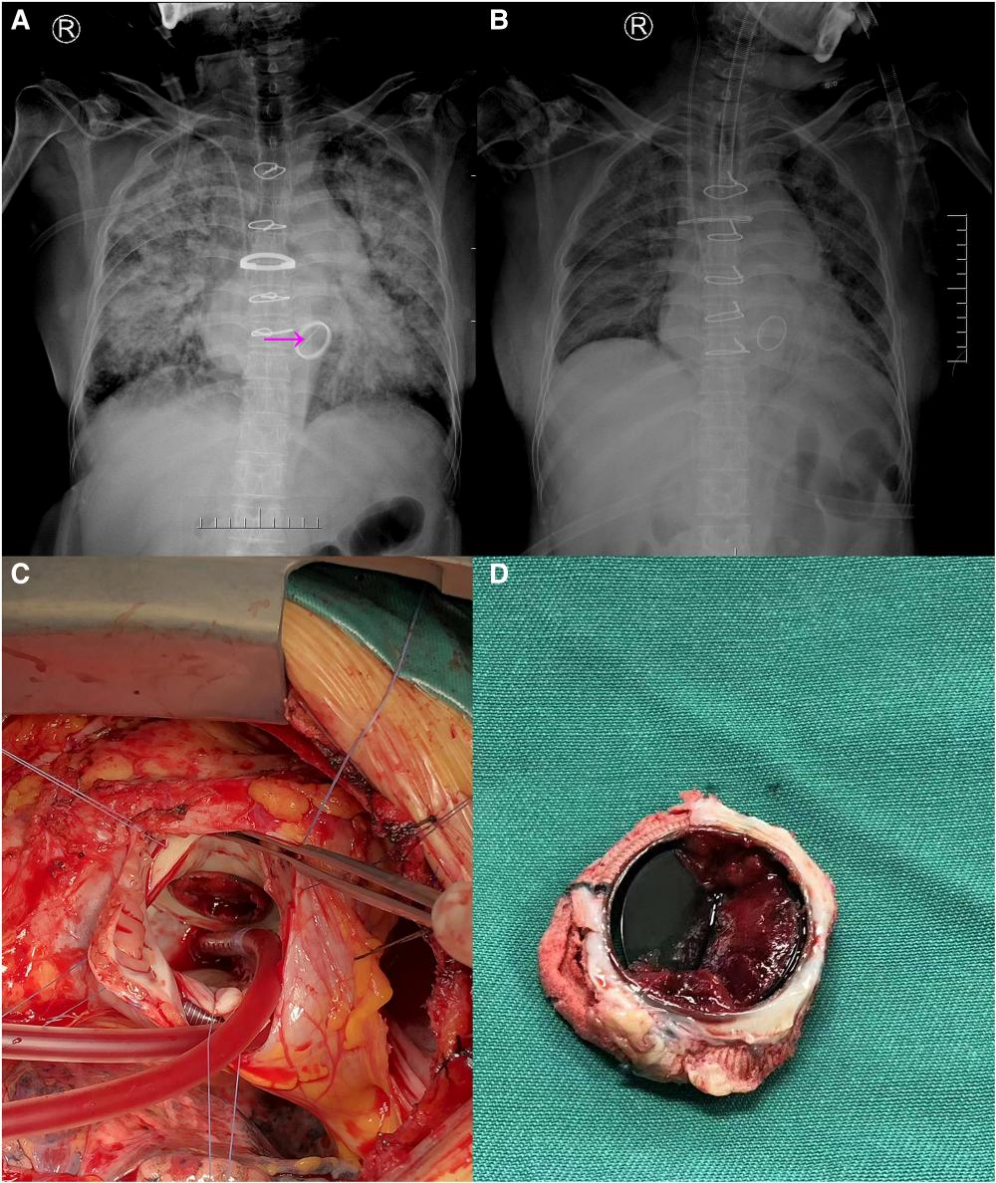
Imaging and surgical findings of acute prosthetic mitral valve obstruction. (*A*) Admission chest X-ray reveals the ‘frozen leaflet sign’ (arrow) and pulmonary venous congestion. (*B*) Postoperative chest X-ray shows resolution of the radiopaque shadow. (*C* and *D*) Intraoperative photographs confirming thrombus causing the valve leaflet obstruction.

A 52-year-old female with a history of mechanical mitral valve replacement (St. Jude Medical Mechanical Valve, 27 mm) for rheumatic heart disease presented with acute decompensated heart failure (NYHA class IV), accompanied by hypotension and hypoxaemia (SpO₂ 79% on room air). A portable anteroposterior chest X-ray revealed pulmonary vascular cephalization and a linear radiopaque density aligned with the prosthetic annulus—a finding we refer to as the ‘frozen leaflet sign’—which raised suspicion for leaflet immobilization (*Figure 1*, panel A, arrow). Transthoracic echocardiography demonstrated elevated transprosthetic gradients (peak/mean 28/16 mmHg), severe pulmonary hypertension (RVSP 65 mmHg), and moderate tricuspid regurgitation. Although her admission INR was 2.03, which is within the lower end of the typical therapeutic range for a mechanical mitral prosthesis, historical INR values were not available. Thus, subtherapeutic anticoagulation prior to admission could not be excluded, which would be consistent with thrombotic obstruction. Acute prosthetic valve obstruction due to thrombosis was strongly suspected based on the clinical and echocardiographic presentation. The patient underwent urgent surgical intervention, which confirmed thrombotic occlusion of the lateral leaflet (*Figure 1*, panels C, D), and successful redo valve replacement was performed. Postoperative radiography showed resolution of the abnormal shadow (*Figure 1*, panel B). This case highlights that the ‘frozen leaflet sign’ on routine CXR, although uncommon and not a validated diagnostic sign, can serve as an early visual clue to acute prosthetic valve obstruction. While fluoroscopy and TEE remain the gold standards for evaluating PVO,^1^ and the absence of this sign does not rule out pathology, its recognition when present on an initial CXR may contribute to early diagnostic suspicion and prompt further investigation.

## Data Availability

No new data were generated or analysed in support of this research.
